# Building Risk Prediction Models for Type 2 Diabetes Using Machine Learning Techniques

**DOI:** 10.5888/pcd16.190109

**Published:** 2019-09-19

**Authors:** Zidian Xie, Olga Nikolayeva, Jiebo Luo, Dongmei Li

**Affiliations:** 1 Clinical and Translational Science Institute, University of Rochester School of Medicine and Dentistry, Rochester, New York; 2 Goergen Institute of Data Sciences, University of Rochester, Rochester, New York; 3 Department of Computer Science, University of Rochester, Rochester, New York

## Abstract

**Introduction:**

As one of the most prevalent chronic diseases in the United States, diabetes, especially type 2 diabetes, affects the health of millions of people and puts an enormous financial burden on the US economy. We aimed to develop predictive models to identify risk factors for type 2 diabetes, which could help facilitate early diagnosis and intervention and also reduce medical costs.

**Methods:**

We analyzed cross-sectional data on 138,146 participants, including 20,467 with type 2 diabetes, from the 2014 Behavioral Risk Factor Surveillance System. We built several machine learning models for predicting type 2 diabetes, including support vector machine, decision tree, logistic regression, random forest, neural network, and Gaussian Naive Bayes classifiers. We used univariable and multivariable weighted logistic regression models to investigate the associations of potential risk factors with type 2 diabetes.

**Results:**

All predictive models for type 2 diabetes achieved a high area under the curve (AUC), ranging from 0.7182 to 0.7949. Although the neural network model had the highest accuracy (82.4%), specificity (90.2%), and AUC (0.7949), the decision tree model had the highest sensitivity (51.6%) for type 2 diabetes. We found that people who slept 9 or more hours per day (adjusted odds ratio [aOR] = 1.13, 95% confidence interval [CI], 1.03–1.25) or had checkup frequency of less than 1 year (aOR = 2.31, 95% CI, 1.86–2.85) had higher risk for type 2 diabetes.

**Conclusion:**

Of the 8 predictive models, the neural network model gave the best model performance with the highest AUC value; however, the decision tree model is preferred for initial screening for type 2 diabetes because it had the highest sensitivity and, therefore, detection rate. We confirmed previously reported risk factors and also identified sleeping time and frequency of checkup as 2 new potential risk factors related to type 2 diabetes.

**Summary**

**What is already known on this topic?**

Many predictive models have been built and many risk factors have been identified for type 2 diabetes. However, previous predictive models based on survey data need to be further improved, and other risk factors need to be identified.

**What is added by this report?**

Our predictive models solely based on survey data performed well in predicting type 2 diabetes and identified 2 additional risk factors for type 2 diabetes.

**What are the implications for public health practice?**

Our findings provide potential useful tools for the initial efficient screening for type 2 diabetes, which can facilitate early intervention and reduce the prevalence of type 2 diabetes.

## Introduction

Diabetes is a chronic disease that increases risk for stroke, kidney failure, renal complications, peripheral vascular disease, heart disease, and death (1). The International Diabetes Federation estimates that by 2045, at the current growth rate, 693 million people will have diabetes worldwide (2). According to the Centers for Disease Control and Prevention (CDC), in 2012, 29.1 million people in the United States were diagnosed with diabetes, making it the seventh leading cause of death in the country (3). Diabetes puts a high financial burden on the US economy. Studies show the total estimated cost of diagnosed diabetes increased to $327 billion in 2017, including $237 billion in direct medical costs and $90 billion in reduced productivity (4).

There are 3 main types of diabetes: type 1, type 2, and gestational. Of those 3, type 2 diabetes is the most prevalent and accounts for 90% to 95% of all cases. Type 2 diabetes is a predictable and preventable disease because it usually develops later in life (age >30) as a result of lifestyle (eg, low physical activity, obesity status) and other (eg, age, sex, race, family history) risk factors (5,6). Many models have been built to predict the occurrence of type 2 diabetes (7–10). However, because of its causal complexity, the prediction performance (especially sensitivity) of models for type 2 diabetes based on survey data needs improvement (11). In addition, although many risk factors, including obesity and age, are well established for type 2 diabetes, others remain to be identified.

To identify the risk factors for a variety of human diseases, in 1984 CDC initiated the state-wide Behavioral Risk Factor Surveillance System (BRFSS), an ongoing, state-based, random-digit–dialed telephone survey of noninstitutionalized US adults aged 18 years or older. The goal of our study was to build predictive models for type 2 diabetes using 2014 BRFSS data by applying machine learning techniques, including support vector machine (SVM), decision tree, logistic regression, random forest, Gaussian Naive Bayes classifiers, and neural network. In addition, we expected to identify other risk factors for type 2 diabetes using statistical methods.

## Methods

### Data source

We accessed publicly available 2014 BRFSS data, which contain 279 variables on 464,644 subjects (https://www.cdc.gov/brfss/annual_data/annual_2014.html). Of these records, 61,118 respondents had been diagnosed with diabetes, 12,699 had been diagnosed with prediabetes, and 390,827 had neither diabetes nor prediabetes. Most of the 279 variables were associated with chronic health conditions other than diabetes (eg, cancer, asthma). A respondent was considered to have type 2 diabetes if the respondent was older than 30, not pregnant, and answered yes to the question “Have you ever been told you have diabetes?”

### Data analysis

According to literature on the risk factors for diabetes (9), we selected 27 variables for analysis (Appendix A). The dependent variable was whether respondents had been told they have diabetes. Respondents younger than 30 years were excluded, because they were most likely to have type 1 diabetes. Respondents who had diabetes and also were pregnant were excluded, as were respondents with prediabetes. The independent variables were general and mental health status; health care coverage and primary source of health care coverage; metropolitan status code; frequency of checkup; exercise; amount of sleep per night; whether they have health problems that require the use of special equipment (eg, cane), are blind or have serious trouble seeing, or have trouble concentrating/remembering/making decisions; whether they were ever told they had angina or coronary heart disease, depression, or kidney disease; flu shot status; smoking status; whether they do physical activity outside of work; marital status; employment status; annual income; whether they own or rent a home; sex; race/ethnicity; age; body mass index (BMI); and level of education completed.

After the variables were selected, any record that had missing values, such as if a subject answered “don’t know” or refused to answer, was excluded from our analysis. We condensed categories for age (31–40 y, 41–50 y, 51–60 y, 61–70 y, 71–80 y, >80 y), mental health (on how many days did you feel depressed over the course of the month: 1–5, 6–30, none), and sleep time (hours per day: 1–6, 7–8, ≥9). We used R software version 3.5.1 (R Foundation) to preprocess the data, and 138,146 respondents (20,467 with type 2 diabetes) were retained.

Several supervised machine learning classifiers have been explored to predict type 2 diabetes using the 2014 BRFSS data set, including SVM (linear, polynomial, and radical basis function [rbf]), Gaussian Naive Bayes, logistic regression, neural network, decision tree, and random forest (12–16). We randomly selected two-thirds of the data from the preprocessed data to be the training data set, with the remaining one-third being the test data set (holdout method). Only 14.8% people had type 2 diabetes in our final data set, so we applied the Synthetic Minority Over-sampling Technique (SMOTE) to avoid model bias (ie, a similar number of people with type 2 diabetes to people without type 2 diabetes) (17). We used R software to construct the predictive models for type 2 diabetes with the SMOTE balanced training data set. The predictive performance of the constructed predictive models was evaluated with the imbalanced test data set using accuracy, sensitivity, and specificity, as well as areas under the receiver operating characteristic (ROC) curves, the AUC value.

We used univariable and multivariable weighted logistic regression models to measure the associations of different variables with type 2 diabetes. To adjust the effect of other variables, the covariates were incorporated into the multivariable weighted logistic regression models. For each variable, we chose one category as the control and calculated odds ratios (ORs) or adjusted odds ratios (aORs) and 95% confidence intervals (CIs) for the other categories. All statistical analyses were conducted using SAS version 9.4 (SAS Institute Inc). Significance for all tests was set at *P* < .05.

## Results

### Predictive models for type 2 diabetes

All classifiers had a high test accuracy (74.3%–82.4%) and high AUC values (0.7182–0.7949) (Table 1). Although the neural network model gave the highest accuracy (82.4%), specificity (90.2%), and AUC (0.7949) values, its sensitivity (37.8%) was the lowest. In contrast, although the decision tree model had the lowest accuracy (74.3%), specificity (78.2%), and AUC (0.7182) values, its sensitivity (51.6%) was the highest. Other classifiers gave intermediate and reasonable accuracy, sensitivity, specificity, and AUC values. Overall, the predictive models for type 2 diabetes had similar and good prediction performance with only slight differences.

**Table 1:** Performance of Predictive Models for Type 2 Diabetes, Data From the Behavioral Risk Factor Surveillance System, 2014

Model	Accuracy	Sensitivity	Specificity	AUC
Neural network	0.8241	0.3781	0.9016	0.7949
Logistic regression	0.8068	0.4634	0.8666	0.7932
Linear SVM	0.8082	0.4260	0.8746	0.7807
Rbf SVM	0.8178	0.4014	0.8902	0.7788
Random forest	0.7927	0.5029	0.8431	0.7608
Naïve Bayes	0.7756	0.4876	0.8256	0.7598
Polynomial SVM	0.7962	0.4515	0.8561	0.7587
Decision tree	0.7426	0.5161	0.7820	0.7182

Abbreviations: AUC, area under the curve; rbf, radical basis function; SVM, support vector machine.

### Risk factors affecting type 2 diabetes

Unadjusted and adjusted ORs from univariable and multivariable weighted logistic regression models are summarized in Table 2. Compared with women, men had a significantly higher risk of type 2 diabetes (aOR = 1.38; 95% CI, 1.29–1.48). Risk of developing type 2 diabetes increased as age and BMI increased, and it decreased as income increased. Compared with married respondents, all other groups had similar risk of developing type 2 diabetes, with the exception of divorced respondents for whom risk of type 2 diabetes was lower. Respondents of all races/ethnicities except Native Hawaiian/other Pacific Islanders had a significantly higher risk of type 2 diabetes than white respondents, and Asians had the highest risk after adjusting for other variables.

**Table 2:** Association Between Covariates With Type 2 Diabetes, Behavioral Risk Factor Surveillance System, 2014

Variable	Unadjusted	Adjusted
	Odds Ratio (95% Confidence Interval)	
**Sex**		
Male	1.30 (1.23–1.37)	1.38 (1.29–1.48)
Female	1 [Reference]	
**Age, y**		
31–40	1 [Reference]	
41–50	3.34 (2.58–4.33)	3.35 (2.56–4.37)
51–60	7.03 (5.54–8.91)	5.81 (4.53–7.46)
61–70	12.41 (9.82–15.67)	8.78 (6.82–11.29)
71–80	16.16 (12.78–20.44)	10.48 (8.05–13.65)
>81	12.71 (9.99–16.17)	8.00 (6.05–10.57)
**Body mass index**		
Normal weight	1 [Reference]	
Underweight	0.88 (0.62–1.25)	0.57 (0.40–0.81)
Overweight	2.18 (2.00–2.38)	1.91 (1.75–2.09)
Obese	5.33 (4.90–5.79)	4.17 (3.81–4.55)
**Annual household income, $**		
<10,000	4.16 (3.55–4.87)	1.56 (1.28–1.90)
10,000–15,000	4.52 (3.99–5.12)	1.47 (1.25–1.73)
15,000–20,000	3.52 (3.16–3.93)	1.27 (1.09–1.47)
20,000–25,000	3.46 (3.14–3.83)	1.38 (1.22–1.57)
25,000–35,000	2.87 (2.60–3.16)	1.27 (1.12–1.44)
35,000–50,000	2.21 (2.20–2.41)	1.18 (1.05–1.31)
50,001–75,000	1.75 (1.60–1.92)	1.17 (1.06–1.30)
>75,000	1 [Reference]	
**Marital status**		
Married	1 [Reference]	
Divorced	1.31 (1.21–1.42)	0.87 (0.79–0.96)
Widowed	2.10 (1.96–2.25)	1.05 (0.96–1.14)
Separated	1.37 (1.13–1.65)	0.82 (0.65–1.02)
Never married	1.16 (1.04–1.30)	1.11 (0.98–1.26)
Member of an unmarried couple	0.86 (0.67–1.11)	1.02 (0.77–1.34)
**Race/ethnicity**		
White only	1 [Reference]	
Black only	1.86 (1.71–2.02)	1.63 (1.47–1.80)
American Indian or Alaskan Native only	2.06 (1.52–2.78)	1.66 (1.07–2.60)
Asian only	0.80 (0.60–1.06)	2.04 (1.51–2.76)
Native Hawaiian or other Pacific Islander only	1.53 (0.52–4.49)	2.66 (0.76–9.31)
Other race only	1.70 (1.16–2.48)	1.79 (1.10–2.92)
Multiracial	1.39 (1.12–1.71)	1.34 (1.05–1.71)
Hispanic	1.26 (1.08–1.47)	1.27 (1.07–1.52)
**Sleep amount, h/d**		
1–6	1.27 (1.20–1.35)	1.03 (0.96–1.11)
7–8	1 [Reference]	
≥9	1.97 (1.80–2.15)	1.13 (1.03–1.25)
**How long since last checkup**		
<1 year	3.75 (2.98–4.72)	2.31 (1.86–2.85)
1–2 years	1.29 (0.99–1.67)	1.20 (0.94–1.54)
3–5 years	1 [Reference]	
>5 years	0.84 (0.62–1.40)	0.82 (0.61–1.10)
Never	1.98 (1.15–3.42)	1.43 (0.80–2.55)

Respondents who slept 6 or fewer, or 9 or more hours per day had a higher unadjusted odds of type 2 diabetes than respondents who slept from 7 to 8 hours per day, but the significance remained only for those who slept 9 or more hours per day after adjusting for all other variables (aOR = 1.13; 95% CI, 1.03–1.25). Compared with respondents whose last checkup was within the last 3 to 5 years, those whose last checkup was less than 1 year ago or who had never had a checkup had higher unadjusted odds of developing type 2 diabetes. After adjusting for all other variables, odds of developing type 2 diabetes were significantly higher only for those whose last checkup was less than 1 year ago (aOR = 2.31; 95% CI, 1.86–2.85). Although the adjusted odds of never having had a checkup were 1.43, they were not significant.

## Discussion

Although many predictive models for type 2 diabetes have been built, most studies have used logistic regression and Cox models (18). In this study, we built predictive models for type 2 diabetes using multiple machine learning algorithms, including SVM, decision tree, logistic regression, neural network, random forest, and Gaussian Naive Bayes. By comparing their prediction performance on the test data set, our predictive models showed similar performance in predicting type 2 diabetes in terms of AUC, sensitivity, specificity, and accuracy. However, the neural network prediction model had the highest accuracy, specificity, and AUC values. In contrast, the decision tree prediction model had the highest sensitivity. 

Other machine learning techniques have similar model performance to logistic regression for predicting type 2 diabetes (19). Although some predictive models performed even better in predicting type 2 diabetes in other studies, with the AUC reaching 0.9, these models were based on longitudinal data sets including clinical data, laboratory measurements, and biomarkers (18). Our decision tree prediction model solely based on national survey data had a 51.6% sensitivity/detection rate, which is an improvement over what was reported by Talmud et al (11), who found a sensitivity/detection rate of 30% to 40% using both survey data and biomarkers. Therefore, our models can provide reasonable initial population screening for type 2 diabetes at a lower data cost, and the decision tree prediction model is preferred because of its high sensitivity/detection rate.

Our statistical analysis was able to confirm well-known risk factors for type 2 diabetes, such as age and BMI, but more importantly may have identified new risk factors. Our analysis showed that not only under sleeping (≤6 hours per day) but also over sleeping (≥9 hours per day) increases risk for type 2 diabetes. It is well known that shorter sleep duration can lead to a higher risk for type 2 diabetes (20), and it has been linked to obesity, glucose intolerance, and insulin resistance (21–23). It has been reported that increases in sleeping time among middle-aged and older women could lead to modestly higher risks of diabetes (24,25). In this study, we demonstrated that the increased risk for type 2 diabetes due to over sleeping applies to all adults. It has been suggested that long sleeping time can have detrimental effects on general health (26), although the mechanism through which this might occur is unknown.

The frequency of getting a checkup is another potential risk factor for type 2 diabetes. Our data showed that getting a checkup within 1 year or never having had checkup increases risk for type 2 diabetes. There are many factors that influence whether a person has a regular checkup. People with diabetes may see a doctor more frequently to monitor their condition, and the lack of a regular doctor visit may be an extension of an unhealthy lifestyle for others. In addition, those who do not have a regular checkup may experience barriers such as lack of transportation or not having health insurance. It is possible that people who do not get a regular checkup missed a possible diagnosis of prediabetes and therefore the opportunity of early intervention and the prevention of type 2 diabetes.

Our study has limitations. Due to the cross-sectional nature of BRFSS data, we could not establish causality. An additional limitation is that BRFSS data were self-reported and subject to recall bias that could affect the performance of our predictive models. However, given the availability of clinical data and biomarkers, our predictive models may perform better in predicting type 2 diabetes. 

We used advanced machine learning techniques to construct predictive models for type 2 diabetes that had good sensitivity and specificity and that helped identify 2 new potential new risk factors for the disease. Our models and findings could allow for early detection, intervention, and prevention of type 2 diabetes.

## Appendix Detailed Information About Selected Variables

**Table:**

Variable	Description	Values
**GENHLTH**	Would you say that in general your health is:	1: Excellent, 2: Very good, 3: Good, 4: Fair, 5: Poor
**X_AGEG5YR**	6 Age categories based on 14 age categories	1: 31 to 40 y, 2: 41–50 y, 3: 51–60 y, 4: 61–70 y, 5: 71–80 y, 6: >81 y
**X_BMI5CAT**	4 Categories of body mass index	1: Underweight, 2: Normal weight, 3: Overweight, 4: Obese
**CHECKUP1**	About how long has it been since you last visited a doctor for a routine checkup?	1: <1 y, 2: 1–2 y, 3: 3–5 y, 4: >5 y, 6: Never
**INCOME2**	What is your annual household income from all sources?	1: <$10 K, 2: $10–$15 K, 3: $15–$20 K, 4: $20–$25 K, 5: $25–$35 K, 6: $35–$50 K, 7: $50–$75 K, 8: >$75 K
**X_RACE**	Race/ethnicity categories	1: White, 2: Black, 3: American Indian or Alaskan Native, 4: Asian, 5: Native Hawaiian or other Pacific Islander, 6: Other race, 7: Multiracial, 8: Hispanic
**MSCODE**	Metropolitan status code	1: Center city, 2: County, 3: Suburban, 5: not in MSA
**FLUSHOT6**	During the past 12 months, have you had either a flu shot or a flu vaccine that was sprayed in your nose?	1: Yes, 2: No
**EMPLOY1**	Are you currently . . .	1: Employed, 2: Self-employed, 3: No work >1 y, 4: No work <1 y, 5: Homemaker, 6: Student, 7: Retired, 8: Unable to work
**SEX**	Indicate sex of respondent	1: Male, 2: Female
**MARITAL**	Are you . . . (marital status)	1: Married, 2: Divorced, 3: Widowed, 4: Separated, 5: Never married, 6: Unmarried couple
**X_EDUCAG**	Level of education completed	1: Did not graduate high school, 2: Graduated high school, 3: Attended college, 4: Graduated college
**SLEPTIM1**	On average, how many hours of sleep do you get in a 24-hour period?	1: 1–6 hours, 2: 7–8 hours, 3: ≥9 hours
**CVDCRHD4**	Have you ever been told you had angina or coronary heart disease?	1: Yes, 2: No
**HLTHCVR1**	What is the primary source of your health care coverage? Is it . . .	1: Employer, 2: Own, 3: Medicare, 4: Medicaid, 5: VA/ Military, 6: Alaska Native or Indian Health Service or Tribal Health Services, 7: Other, 8: None
**MENTHLTH**	Now thinking about your mental health, which includes stress, depression, and problems with emotions, for how many days during the past 30 days was your mental health not good?	1: 0–5, 2: 6–30
**CHCKIDNY**	Have you ever been told you have kidney disease? Do not include kidney stones, bladder infection or incontinence.	1: Yes, 2: No
**USEEQUIP**	Do you now have any health problem that requires you to use special equipment, such as a cane, a wheelchair, a special bed, or a special telephone?	1: Yes, 2: No
**X_TOTINDA**	Adults who reported doing physical activity or exercise during the past 30 days other than their regular job	1: Had physical activity or exercise, 2: No physical activity in past 30 days
**ADDEPEV2**	Have you ever been told you that you have a depressive disorder, including depression, major depression, dysthymia, or minor depression?	1: Yes, 2: No
**RENTHOM1**	Do you own or rent your home?	1: Own, 2: Rent, 3: Other
**EXERANY2**	During the past month, other than your regular job, did you participate in any physical activities or exercises such as running, calisthenics, golf, gardening, or walking for exercise?	1: Yes, 2: No
**BLIND**	Are you blind or do you have serious difficulty seeing, even when wearing glasses?	1: Yes, 2: No
**DECIDE**	Because of a physical, mental, or emotional condition, do you have serious difficulty concentrating, remembering, or making decisions?	1: Yes, 2: No
**HLTHPLN1**	Do you have any kind of health care coverage, including health insurance, prepaid plans such as health maintenance organizations, or government plans such as Medicare or Indian Health Service?	1: Yes, 2: No
**DIABETE3**	Have you ever been told you have diabetes?	1: Yes, 2: Yes but pregnant, 3: No, 4: Prediabetes
**_SMOKER3**	4 Levels of smoking status	1: Current smoker every day, 2: Current smoker some days, 3: Former smoker, 4: Never smoked
